# Deficiencies in social relationships of individuals with neurosis

**DOI:** 10.4103/0019-5545.31578

**Published:** 2006

**Authors:** Sunil Srivastava

**Affiliations:** * Senior Psychiatrist, Institute of Mental Health and Hospital, Agra, Uttar Pradesh; e-mail: sunilsrivastava99@gmail.com

**Keywords:** Primary group, neurosis, social interaction

## Abstract

**Background:**

Social interaction and network of individuals with neurosis have been reported to be inadequate.

**Aim:**

To measure deficiencies in the social network of individuals with neurosis.

**Methods:**

Fifty consecutive patients with neurosis attending the OPD of the Department of Psychiatry, King George's Medical College, Lucknow, who were diagnosed as per the criteria of ICD-9, were included in the study. A control group of 40 healthy persons matched for age, sex, education and marital status was also taken. Interaction with the primary group (defined as consisting of all kin, nominated friends, work associates and neighbours) was measured by the Social Interaction Schedule of Henderson *et al.* as modified for the Indian population.

**Results:**

Patients reported significantly higher mean duration of unpleasant but affectively intense interaction with one person within or outside the primary group and affectively unpleasant and intense interaction with more than one person within or outside the primary group or affectively superficial but unpleasant interaction with one or more person of the primary group. Healthy subjects reported more of pleasant interaction with one person within or outside the primary group and affectively intense and pleasant interaction with more than one person within or outside the primary group or affectively superficial but pleasant interaction with one or more persons of the primary group; the difference was statistically significant as compared to patients with neurosis.

**Conclusion:**

The primary group of patients with neurosis was significantly smaller in numerical size as compared with that of controls and in terms of the total time spent with members of the primary group, patients with neurosis reported more interactions of unpleasant type and less of pleasant type as compared with healthy controls.

## INTRODUCTION

It is important to study the social relationships of individuals with neurosis because a person's ability to establish and maintain relationships may be impaired by the presence of psychiatric illness or social relationship may serve as a protective function against psychiatric morbidity in the face of adverse life experiences.^1^–^6^ The lack of social relationships has also been proposed as a causal factor in the development of neurotic disorders independent of adverse life experiences.^7^,^8^ Investigators have found that psychologically distressed persons have less rewarding social network and social ties.^9^–^11^ Negative correlations between social support and stress and dysfunction has been demonstrated by Indian researchers too.^12^ In psychiatric epidemiology, particularly in relation to neurosis, some of the observed differentials in prevalence rates can partly be explained by deficiencies in social bonds.^13^–^15^ The relevance and need for a systematic study of social relationship and neurosis is, therefore, self-evident. There is a large literature on social networks but qualitative analysis of interaction has not been given much attention; instead the structure of relationships or the composition of the primary group has received more attention.^16^–^20^ These studies, therefore, reveal little of what is psychologically important in social relationships.

The most important component of social environment of an individual is his ‘primary group’; a concept introduced by Cooley^19^ and defined concisely by Broom and Selznick^21^ as immediate social network formed by individuals with whom one has interaction and commitment. As is evident from the definition, the boundaries of primary group are not hard and fast but mainly consist of household members, neighbours, close relatives and good friends. The group, being persistent and close, is important for the psychological health of an individual as social support in the face of stress is mediated through the primary group and psychiatric illness is likely to have an early effect on a person's relationships with members of the primary group.

The present study was designed to find out deficiencies, if any, in the structure of primary group and interaction of individuals with neurosis as measured on duration, intensity and affective quality of interaction with members of the primary group in comparison to healthy controls.

## METHODS

An index group of 50 consecutive patients with neurosis who fulfilled the inclusion and exclusion criteria was taken from the OPD of the Psychiatry Department of King George's Medical College, Lucknow. One inclusion criterion was that the patient should be diagnosed to be suffering from a neurotic disorder as per the ICD-9.^22^ Exclusion criteria included the presence of a concurrent physical illness, education less than class VIII and rural domicile. Minimum education up to class VIII was ensured so that subjects did not have any difficulty in following the Social Interaction Schedule used in this study. Patients residing in rural areas were excluded because interaction via telephone is also recorded by the instrument of the study, and this could have seriously affected the homogeneity of the sample. A control group of 40 healthy subjects of urban domicile was taken matched on the parameters of age, sex, education and marital status with that of the index group.

The subjects in the index group were first administered a semi-structured proforma to record identification data, sociodemographic characteristics and findings of psychiatric evaluation. Similarly, identification data and sociodemographic characteristics were recorded on a preset proforma for subjects of the control group and presence of psychiatric illness was excluded by a clinical examination and evaluation on Cornell Medical Health Questionnaire.^23^

The operational definition of primary group, as given by Henderson *et al*.^1^ has been taken, which states that ‘the primary group of a person is a group made up of all his kin, nominated friends, work associates and neighbours’. To measure the interaction with the primary group for both the patients with neurosis and subjects of the control group, Modified Social Interaction Schedule of Henderson *et al.*^8^ was administered. The schedule was developed by Henderson *et al.*^8^ and was modified for the Indian population by Sethi *et al*.^24^,^25^ It identifies subject's primary group and examines interaction with members of the primary and non-primary groups. The schedule first records numerical size and composition of an individual's household members, number of all other kin in the community considered by respondent as close relative, and persons whom he or she considers as good friends.

The interview then evaluates details of the subject's interaction 7 days before the interview regarding the person or persons with whom he or she interacted, contents of interaction, whether it was affectively intense or superficial, the duration of interaction, mode of interaction, i.e. direct, on telephone or by letter, and whether the subject considered the interaction as pleasant, unpleasant or neutral. Duration of interaction was recorded in the intervals of 5 minutes with minimum interaction taken as of 2 minutes. The interactions have been divided into three types as follows:

**Table:** 

Type-I:	Affectively intense interaction with one person only which may be within or outside the primary group.
Type-II:	Affectively intense interaction with more than one person within or outside the primary group or affectively superficial interaction with one or more persons who are within the primary group.
Type-III:	Affectively superficial interaction with one or more persons who are outside the primary group.

Intensity of interaction was not defined and it was based totally on subject's impression. Chi-square test was used to determine any significant difference between the composition of the index and control groups. Student *t*-test was applied to evaluate significant differences in the means between the index and control groups on variables of size of the primary group, duration of interaction and quality of interaction.

## RESULTS

Table 1 shows the distribution of patients and controls matched for age, sex and marital status. Chi-square test did not show any statistically significant difference between the two groups on these parameters. Majority of the patients were unmarried (56%), males (58%) and of the age group 21–30 years (50%). Table 2 shows that the index sample consisted of patients mainly of neurotic depression (38%), anxiety state (30%) and hysteria (26%) while patients of obsessive–compulsive disorder (OCD) were few (6%).

**Table 1:** Sample composition in relation to age, sex and marital status

	Patients (*n*=50)		Controls (*n*=40)	
		
	*n*	(%)	*n*	(%)
Age in years				
Up to 20	17	34	14	35
21-30	25	50	19	47.5
31-40	5	10	5	12.5
41 and above	3	6	2	5
Sex				
Male	29	58	23	57.5
Female	21	42	17	42.5
Marital Status				
Married	22	44	19	47.5
Unmarried	28	56	21	52.5
Widow	-	-	-	-
Separated	-	-	-	-

For age *X^2^*=0.06, not significant; for sex *X^2^*=0.002, not significant; for marital status *X^2^*=0.01, not significant.

**Table 2:** Break-up of the index sample on diagnosis

Diagnosis	Patients (*n*=50)	
	
	*n*	%
Anxiety state	15	30
Hysteria	13	26
Obsessive-compulsive disorder	3	6
Neurotic depression	19	38

Table 3 shows the data on the size of the primary group; the numerical size of household members of patients was smaller (p<0.05) as compared with that of the control group. Further, patients had significantly lesser number of close relatives (p<0.01) and good friends (p<0.001) as compared with controls.

**Table 3:** Sample break-up on primary-group size

	Patients (*n*=50)		Controls (*n*=40)		
			
Primary group subcategory	Mean	SD	Mean	SD	
Number of household members	5.4	1.1	5.9	1.2	t=2.05, p<0.05
Number of close relatives	1.6	0.4	1.9	0.5	t=2.72, p<0.01
Number of good-friends	2.5	0.4	3.1	0.6	t=4.85, p<0.001

For all comparisons df=88

Table 4 presents mean and standard deviation of the total duration of interaction in hours in the index week of patients and controls with members of the primary subgroups or non-primary group. The patients did not differ significantly with the control group in terms of total interaction with household members, total interaction with primary group members other than household members, and in total interaction outside the primary group. Table 4 shows that mean duration of Type-I positive (pleasant) interaction of patients was significantly less than that of controls (p<0.01), whereas mean duration of interaction of Type-I negative (unpleasant) interaction was higher in the patients (p<0.001). Patients reported significantly lower Type-II positive interaction (p<0.001) in comparison with controls, but patients had a higher Type-II negative interaction as compared with healthy controls (p<0.001). No significant difference was found between the index group and the control group on Type-III interaction, i.e. affectively superficial interaction with one or more persons who are outside the primary group.

**Table 4:** Break-up of total interaction (in hours) during the index week

	Patients (*n*=50)		Controls (*n*=40)		
			
	Mean	SD	Mean	SD	
Total interaction with household members	15.8	3.1	16.3	2.8	NS
Total interaction with primary group members other than household members	4.8	1.3	4.91	1.3	NS
Total interaction with persons outside the primary group	9.2	1.6	8.86	2.1	NS
Total Type-I positive interaction	2.9	0.3	3.1	0.23	t=2.78, p<0.01
Total Type-I negative interaction	2.5	0.6	1.7	0.4	t=7.3, p<0.001
Total Type-I neutral interaction	2.2	0.9	2.4	1.2	NS
Total Type-II positive interaction	4.7	1.6	7.4	2.1	t=6.9, p<0.001
Total Type-II negative interaction	4.6	1.1	3.3	1.0	t=5.79, p<0.001
Total Type-II neutral interaction	8.8	2.3	8.3	2.2	NS

For all comparisons df=88; NS: not significant

Table 5 shows qualitative break-up of interaction with members of the primary group both for the index group and the control group. The control subjects had significantly higher mean duration of Type-II positive interaction with household members as compared with patients (p<0.01) and the patients differed significantly on mean duration of Type-II negative interaction with household, which was higher in the index group in comparison with controls (p<0.01). On Type-I unpleasant interaction with household members, mean duration of Type-I negative interaction for the control subjects was significantly less as compared with that of patients (p<0.05). On interactions with members of the primary group outside household the patients reported more of Type-I negative interaction in comparison with controls; the difference was highly significant (p<0.001). No significant difference in interaction with persons outside the primary group between the index group and control subjects was observed.

**Table 5:** Break-up of primary group interaction (in hours) during the index week

	Patients (*n*=50)		Controls (*n*=40)		
			
	Mean	SD	Mean	SD	
Interaction with household members				
Type-I positive	1.5	0.3	1.6	0.3	NS
Type-I negative	1.6	1.6	1.3	0.6	t=2.35, p<0.05
Type-I neutral	1.5	0.5	1.7	0.6	NS
Type-II positive	2.7	2.6	4.5	3.0	t=3.04, p<0.01
Type-II negative	3.2	2.8	1.9	1.9	t=2.88, p<0.01
Type-II neutral	4.7	0.8	4.9	0.7	NS
Interaction with primary group members other than household members				
Type-I positive	0.75	0.4	0.9	0.5	NS
Type-I negative	0.3	0.1	0.1	0.09	t=9.8, p<0.001
Type-I neutral	0.3	0.06	0.31	0.08	NS
Type-II positive	1.5	1.2	1.8	1.5	NS
Type-II negative	0.4	0.4	0.3	0.3	NS
Type-II neutral	1.2	0.6	1.4	0.6	NS

For all comparisons df=88; NS: not significant

On inter-diagnostic group comparison on the quantity and quality of interaction between anxiety state and hysteria, between anxiety state and neurotic depression, and between neurotic depression and hysteria no statistically significant difference was found. The OCD group was excluded from comparison as it was thinly represented.

## DISCUSSION

In patients with neurosis, a causal relation of inadequacy of or pathological interaction with social network has been pointed out by several workers.^8^,^26^ In the present study, it was observed that the primary group of the patients was numerically smaller as compared with that of the controls. Henderson *et al.*^8^ also reported a similar observation. Sethi *et al.*^25^ did not find any significant difference between the size of the primary group of patients with neurosis in comparison to healthy controls (which might have been due to the smaller sample size of the study as realized by the authors too). Implications of this finding are difficult to analyse and it can be interpreted either way. Smaller number of close relatives and of good friends may be causally related to neurosis as reported by other observers or it may be due to altered perception of the patient due to the illness or due to the subject's inability to enter into or maintain relationships due to morbidity.^3^,^4^,^7^ However, the third component of the primary group, i.e. number of household members, is likely to have a contributory effect on the development of neurosis because the numerical size of household members is less likely to be influenced by the altered perception though it is likely to be influenced by the behavioural illness of the patient.

In respect of affective quality of interactions, healthy subjects reported more of Type-II pleasant interaction with household members. In patients, significantly higher mean duration of Type-I unpleasant interaction with household members and persons outside household but within the primary group was reported. Simply stated, patients reported significantly more of their interaction as unpleasant with members of the primary group as compared to controls. Again, it may be a primary influencer related to the subsequent development of neurotic illness or it may be a secondary influencer due to changed perception as a symptom of the illness. As intra-group comparison in subjects of the index group between different neurotic illnesses did not reveal any significant difference, the finding appears more likely to have a causal influence on the neurotic illness because cognitive distortions vary in different neurotic illnesses. Sethi *et al.*^25^ did not find any deficiency in the affective quality of interaction of patients with members of the primary group in comparison with control, being contrary to findings of Henderson *et al.*^8^ and which they felt could have been due to social desirability and reticence in openly discussing family problems. These observations lend support to the concept that persons who view their relationships as inadequate have an increased risk of developing neuroses.^27^–^29^

## CONCLUSION

The study shows that the primary group of patients with neurosis was significantly smaller in size as compared with controls. Of the total time spent with members of the primary group, interaction of patients were more of unpleasant type and less of pleasant type in comparison with healthy controls. A major limitation of the study is that it is a retrospective study based on the reporting based on recall of patients. Neurotic illnesses are known to colour perception and influence recall varying from forgetting to selective attention. Secondly, the week prior to the day of observation for interactional analysis may be more representative of the perception of the patient and is less likely to display interactional patterns leading to the development of illness. A period prior to onset of illness is more likely to be of aetiological significance but that could not be taken for the reason of poor recollection by the subjects. A prospective study on a large healthy population for such a period as to have statistically analyaable size of patients with neurosis using similar tools, though difficult, would overcome these limitations and is therefore desirable.
